# Uncovering a Vital Band Gap Mechanism of Pnictides

**DOI:** 10.1002/advs.202105787

**Published:** 2022-03-31

**Authors:** Jindong Chen, Qingchen Wu, Haotian Tian, Xiaotian Jiang, Feng Xu, Xin Zhao, Zheshuai Lin, Min Luo, Ning Ye

**Affiliations:** ^1^ Key Laboratory of Optoelectronic Materials Chemistry and Physics Fujian Institute of Research on the Structure of Matter Chinese Academy of Sciences Fuzhou Fujian 350002 China; ^2^ Tianjin Key Laboratory of Functional Crystal Materials Institute of Functional Crystal Tianjin University of Technology Tianjin 300384 China; ^3^ Technical Institute of Physics and Chemistry Chinese Academy of Sciences Beijing 100190 China; ^4^ State Key Laboratory of Physical Chemistry of Solid Surfaces Collaborative Innovation Centre of Chemistry for Energy Materials College of Chemistry and Chemical Engineering Xiamen University Xiamen 361005 China; ^5^ University of Chinese Academy of Sciences Beijing 100049 China

**Keywords:** band gap, electronic structure, infrared NLO materials, pnictides

## Abstract

Pnictides are superior infrared (IR) nonlinear optical (NLO) material candidates, but the exploration of NLO pnictides is still tardy due to lack of rational material design strategies. An in‐depth understanding structure–performance relationship is urgent for designing novel and eminent pnictide NLO materials. Herein, this work unravels a vital band gap mechanism of pnictides, namely P atom with low coordination numbers (2 CN) will cause the decrease of band gap due to the delocalization of non‐bonding electron pairs. Accordingly, a general design paradigm for NLO pnictides, ionicity–covalency–metallicity regulation is proposed for designing wide‐band gap NLO pnictides with maintained SHG effect. Driven by this idea, millimeter‐level crystals of MgSiP2 are synthesized with a wide band gap (2.34 eV), a strong NLO performance (3.5 x AgGaS_2_), and a wide IR transparency range (0.53–10.3 µm). This work provides an essential guidance for the future design and synthesis of NLO pnictides, and also opens a new perspective at Zintl chemistry important for other material fields.

## Graphical abstract

A crucial band gap mechanism of pnictides is revealed by combining first‐principles calculation with classical Zintl−Klemm concept. A new wide‐bandgap NLO pnictide design paradigm i.e. balancing the ionic–covalent nature and decreasing the metallicity is proposed. Driven by this paradigm, crystals of MgSiP_2_ with ionic–covalent–metallic nature regulation are preliminarily grown as an IR NLO material candidate.

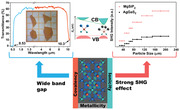

## 1 Introduction

Infrared (IR) nonlinear optical (NLO) materials, capable of generating a coherent IR laser through frequency conversion, play an indispensible role in medical diagnostics, atmospheric detection, laser weapons, and laser telecommunications.^[^ ^1^ ^]^ Currently, the applications of commercial IR NLO materials, AgGaS_2_, AgGaSe_2_, and ZnGeP_2_, are very limited due to their intrinsic defects, e.g., low laser damage thresholds (LDT) of AgGaS_2_, non‐phase‐matching behavior of AgGaSe_2_, and strong two‐photon absorption of ZnGeP_2_.^[^ ^2^ ^]^ Therefore, it is indispensible to design new NLO crystals with large SHG effect, high LDT, suitable band gap, and moderate birefringence.

Chalcogenides and pnictides are both distinguished IR NLO material candidates. Over the past decades, chalcogenide NLO crystals have been widely investigated, and developed numerous state‐of‐the‐art IR NLO materials like BaGa_4_S_7_, BaGa_4_Se_7_, BaGa_2_GeS_6_, BaGa_2_GeSe_6_, LiM^III^X_2_ (M^III^ = Ga and In; X = S and Se) and AgGaGe_n_S_2(n+1)_ (*n* = 2, 3, 4, and 5), etc.^[^ ^3^ ^]^ In recent years, the exploration on pnictide NLO crystals has emerged after years of silence. Compared with chalcogenide NLO crystals, pnictide those, especially diamond‐like pnictides, have natural advantages as NLO materials because they have larger second‐order NLO coefficients and higher thermal conductivity due to larger micro second‐order NLO susceptibility of [MPn_4_] (M = metal, Pn = P, As) tetrahedra and weaker phonon anharmonicity, respectively.^[^ ^4^ ^]^ In addition, pnictides have good IR transmittance and wide transparency range generally exceeding 10 µm. These merits make them promising in high powerful laser output at long‐wave (8–14 µm) IR region. Unfortunately, most of pnictides suffer narrow band gap (<2.33 eV), which means they cannot be efficiently pumped with widely available 1 µm laser sources due to serious two‐photon absorption and free carrier absorption, limiting their practical applications. In addtion, too narrow band gap is also unfavourable to the LDT because they are generally positively related. Therefore, broadening the band gap meanwhile maintaining a large SHG effect becomes crucial but challenging in pnictide NLO material design. In general, the band gap of chalcogenides and oxides can be effectively widen by introduction of strongly electropositive alkali (Li, Na, K, Rb, and Cs) and alkaline earth metals (Mg, Ca, Sr, and Ba). The combination of highly ionic metals and SHG‐active groups e.g. planar trigonal groups ([BO_3_], [B_3_O_6_], [CO_3_], and [NO_3_])^[^ ^5^ ^]^ and tetrahedra groups ([SO_4_], [PO_4_], [PS_4_], [GaS_4_], and [GeS_4_])^[^ ^6^ ^]^ has brought out a plethora of excellent NLO crystals.

**Figure 1:**
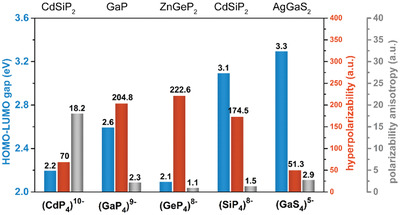
HOMO‐LUMO band gap, hyperpolarizability, and polarizability anisotropy of (CdP_4_)^10−^, (GaP_4_)^9−^, (GeP_4_)^8−^, (SiP_4_)^8−^, and (GaS_4_)^5−^ from diamond‐like pnictides CdSiP_2_, GaP, ZnGeP_2_, CdSiP_2_, and AgGaS_2_, respectively.

Unexpectedly, this material design paradigm cannot work well on increasing the band gap of pnictides. Most multicomponent phosphides containing heavier alkali (Na, K, Rb, and Cs), alkaline earth metals (Ca, Sr, and Ba), and IB, IIB, IIIA, and IVA elements, do not exhibit wider band gap, e.g. BaIn_2_P_2_ (0.4 eV), BaGe_2_P_2_ (1.36 eV), BaGe_7_P_12_ (1.6 eV), Ba_3_Ga_3_P_5_ (1.25 eV), Ba_4_AgGa_5_P_8_ (1.4 eV), Ba_2_SiP_4_ (1.45 eV), Sr_2_SiP_4_ (1.41 eV), Ba_2_Si_3_P_6_ (1.88 eV), *β*‐Ca_2_CdP_2_ (1.55 eV), Ba_5_Ga_6_GeP_12_ (1.39 eV), CaCd_2_P_2_ (1.78 eV), Ba_2_ZnP_2_ (0.6 eV), NaCd_4_Pn_3_ (1.2 eV), NaSnP (0.51 eV), SrSi_7_P_10_ (1.1 eV), BaSi_7_P_10_ (1.1 eV), and KSi_2_P_3_ (1.72 eV).^[^ ^7^, ^8^, ^9^ ^]^ One could simply attribute the reason to the weaker electronegativity of P/As element than S/Se element. Nevertheless, there exist some deeper factors influencing the band gap of pnictides. For instance, our group's previous works have revealed the highly polymerized Ge–Ge homocationic bond with certain metallicity may decrease the band gap of pnictides.^[^ ^7b^ ^]^ However, the reason why alkali/alkaline earth metals pnictides without homocationic bond frequently exhibit the narrow band gaps are still unclear, and to our knowledge, no related studies are reported. Generally, the system of stoichiometric phosphides composed of IA/IIA and IIB/IIIA/IVA element can be divided into anionic framework constructed by tetrahedron groups [MP_4_], and counter cations IA^+^/IIA^2+^. Accordingly, the HOMO‐LUMO band gap, hyperpolarizability and polarizability anisotropy of familiar SHG‐active tetrahedral anionic groups, (CdP_4_)^10−^, (GaP_4_)^9−^, (GeP_4_)^8−^, (SiP_4_)^8−^, and (GaS_4_)^5−^ were investigated. As presented in **Figure** **1**, the [SiP_4_]^8−^ group exhibited considerably wide HOMO‐LUMO band gap of 3.1 eV comparable to (GaS_4_)^5−^ (3.3 eV), and large hyperpolarizability, indicating phosphidosilicates might be most protential NLO material candidates with band gap exceeding or close to 2.33 eV meanwhile strong NLO effect. Unfortunately, a substantial percentage of alkali/alkaline earth metal phosphidosilicates have narrow band gap (<2.0 eV), e.g. SrSi_7_P_10_ (1.1 eV),^[^ ^9d^ ^]^ KSi_2_P_3_ (1.72 eV),^[^ ^9e^ ^]^ Ba_2_SiP_4_ (1.45 eV),^[^ ^8a^ ^]^ Sr_2_SiP_4_ (1.41 eV),^[^ ^8a^ ^]^ Ba_2_Si_3_P_6_ (1.88 eV).^[^ ^8b^ ^]^ In contrast, those pnictides not containing alkali/alkali earth metals or containing halogens or lighter alkali/alkali earth metals Li and Mg, exhibit wider band gap such as GaP (2.4 eV), Zn_3_PI_3_ (2.85 eV), Cd_3_PI_3_ (2.44 eV), and Cd_2_PCl_2_ (>2.5 eV), Li_3_AlP_2_ (2.75 eV), and MgSiP_2_ (2.33 eV).^[^ ^10^ ^]^ Pnictides are generally classified into Zintl compounds and obey the electron count rule^[^ ^11^ ^]^ describing that the electropositive cation donates its valence electrons to a negatively charged polyanion network for which the octet closed shell is fulfilled. Then, why the increase of electronegativity difference between cation and polyanion don't lead to wider band gap in stoichiometric phosphides containing heavier alkali (Na, K, Rb, and Cs) and alkaline earth metals (Ca, Sr, and Ba) like doing in chalcogenides and oxides? It is imperative to reveal the unknown underlying law influencing the band gap of pnictides.

Aiming at getting sight into this band gap mechanism and guiding the design of wide‐band gap NLO pnictides, we investigated the A^II^‐Si‐Pn_2_ family and it structural variation of A^I^_2_‐Si‐Pn_2_ family (A^I^ = Li, Na, K, Rb and Cs; A^II^ = Cd, Mg, Ca, Sr; Pn = P and As).^[^ ^12^, ^13^ ^]^ A abnormal ‘‘band gap reduction’’ was observed among them. MgSiP_2_, Li_2_SiP_2_, and CdSiP_2_ exhibited wider calculated band gap of 2.41, 2.32, and 2.13 eV, and the band gap decreases from 2.41 eV of MgSiP_2_ to 1.81 eV of Cs_2_SiP_2_ with the increase of electropositivity and atomic radius of A site. Via combining first‐principles calculation and classical Zintl‐Klemm concept, the band gap mechanism of alkali/alkali earth pnictides was revealed. A new idea designing wide‐band gap NLO pnictides, i.e. ionic–covalent–metallic nature control, was proposed based on this mechanism. Furthermore, we synthesized millmeter‐level crystals of MgSiP_2_, which has reported largest band gap of 2.34 eV among chalcopyrite pnictides, and simultaneously exhibited strong SHG effects and wide IR range, indicating it is a promissing NLO material candidates.

## 2 Results and Discussion

**Figure 2:**
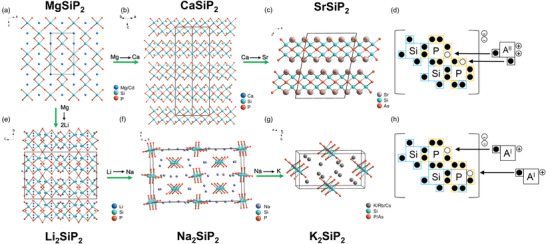
Crystal structure evolution of the title compounds (a–c, e–f) and charge transfer model of A^II^‐Si‐Pn_2_ (d) and A^I^_2_‐Si‐Pn_2_ (h).

MgSiP_2_ and CdSiP_2_ have classical chalcopyrite structure (I‐42d) with three‐dimensional (3D) covalent framework consisting of [SiP_4_] tetrahedra, and Mg/Cd cations fill the tunnels coordinated with four nearest P atoms (**Figure** **2**a). As the increase of the ionicity and radius of A site cation, the ‘‘dimensional reduction’’ effect^[^ ^14^ ^]^ increases and 3D Si‐P framework gradually collapses. CaSiP_2_ (Figure 2b) and SrSiAs_2_ (Figure 2c) have 2D layered (SiPn_2_)^2−^ network, and Ca^2+^ and Sr^2+^ locate on interlayer as counter cations. Although Li_2_SiP_2_ (Figure 2e) holds the 3D Si‐P network, the layered tendency begins to emerge featuring the decreasing Si‐P‐Si connections along b‐axis. The structure of Na_2_SiP_2_ (Figure 2f), K_2_SiP_2_, Rb_2_SiAs_2_, and Cs_2_SiP_2_ (Figure 2g) become one‐dimensional with (SiPn_2_)^2−^ chains and counter ions. Meanwhile, the variety of A site cation creates different coordination environment of Pn atoms. In order to make a qualitative and intuitive comparison, the title phosphides are emphatically investigated (Figure S1, Supporting Information). In MgSiP_2_ and CdSiP_2_, the P atoms are all connected with nearest neighbor two Si atoms and two Mg/Cd atoms, and Mg/Cd and Si atoms are tetrahedrally coordinated with four P atoms, so their structure can be viewed as 3D tetrahedral covalent framework, where the bonding interaction between Mg/Cd and P atoms is dominated by covalency. The P atoms in Li_2_SiP_2_ have four near Li with two shorter Li‐P distance and two longer Li‐P distance while all Li atoms are coordinated tetrahedrally with four P atoms (Figure S2, Supporting Information). Due to the stronger electropositive and larger size of their A site atom in CaSiP_2_, Na_2_SiP_2_, K_2_SiP_2_, and Cs_2_SiP_2_, the A‐P bonding interaction is mainly nondirected and unsaturated electrostatic force. According to Zintl−Klemm concept,^[^ ^11^, ^21^ ^]^ the anionic framework in their structures should achieve an octet closed shell by a complete charge transfer from the electropositive to the electronegative component. Due to the absence of homoatomic bonds, the formal chemical formula can be expressed as A^2+^(Si^4+^)(P^3−^)_2_ or (A^+^)_2_(Si^4+^)(P^3−^)_2_. P atoms accept two electrons from nearest neighbor Si atoms forming two Si‐P covalent bonds. The residual charge on P^3−^ was provided by near A site atoms, making two lone electron pairs under ideal condition (Figure 2d,h). To confirm this charge distribution model, the electron localization function of title phosphides was performed.

**Figure 3:**
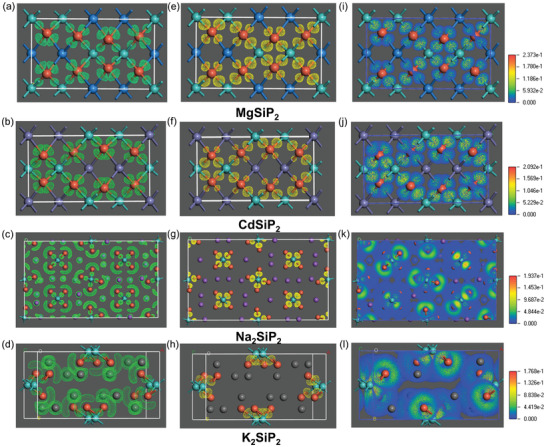
Three‐dimensional (3D) electron localization function (ELF) isosurfaces at *η* = 0.5 (left column), electron density difference (EDD) isosurfaces at *η* = 1/2 × maximum (middle column) and EDD distributions (right column) of MgSiP_2_ (a, e, and i), CdSiP_2_ (b, f, and j), Na_2_SiP_2_ (c, g, and k), and K_2_SiP_2_ (d, h, and l).

**Figure 4:**
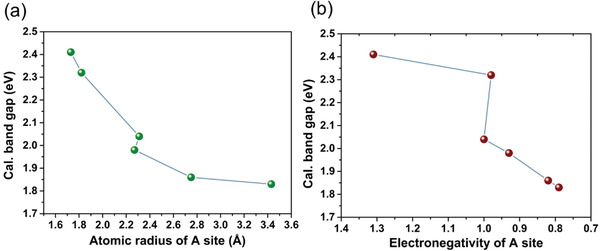
The relationship between cal. band gaps and atomic radius (a) or electronegativity (b) of A site atoms. Not containing CdSiP_2_.

The electron localization function (ELF) maxima of the Si‐P bonds for title compounds are almost halfway between Si and P atoms, and show high localization, which indicates strong covalent character between them. In the ELF plots of MgSiP_2_ and CdSiP_2_ (**Figure** **3**a,b), there are two isolated attractors between one P atom and two near Mg/Cd atoms, indicating the chemical bonding of Mg/Cd‐P can be defined as a polar covalent bond with the localized attractors shifting to P atoms. In Li_2_SiP_2_ (Figure S3a, Supporting Information), there is localized and coherent attractors on P atom toward two nearer Li, indicating certain covalency of shorter Li‐P bonds. Whereas, the ELF maxima of P atoms toward A cations (A = Ca, Na, K, and Cs) become dispersive with larger parallel‐spin pair probability volume indicates nonbonding electron pairs behavior, and ELF distribution around the A site cations (Figure 3c,d and Figures S4 and S5, Supporting Information) is almost spherical which is typical for nondirected electrostatic interactions between A cations and anions.^[^ ^15^ ^]^ Usually, the electronic structure of compounds determine their physical properties. Accordingly, their differential electronic states might cause distinguishing band gap. Herein, the electronic structure of title compounds was calculated based on HSE06 exchange‐correlation functional.^[^ ^16^ ^]^ As shown in **Table** **1**, MgSiP_2_ exhibits widest calculated band gap of 2.41 eV and CdSiP_2_ has band gap of 2.23 eV, which is coincident with their experimental value of 2.33 and 2.15 eV.^[^ ^10^, ^17^ ^]^ Li_2_SiP_2_ has second largest band gap of 2.38 eV accordant with its yellow crystal color.^[^ ^13d^ ^]^ The calculated band gap of CaSiP_2_ is 2.04 eV corresponding to its red crystal color,^[^ ^12c^ ^]^ and that of Na_2_SiP_2_ is 1.98 eV matching up with its dark red crystal color.^[^ ^12b^ ^]^ The band gap of title phosphides declines from 2.41 to 1.83 eV with the variety of atomic radius and electropositivity of A site cations from Mg^2+^ to Cs^+^ (**Figure** **4**). Counterintuitively, the introduction of Ca, Sr, Na, K, Rb, and Cs with stronger ionicity did not broaden but decrease the band gap. By comparing the ELF pattern, it can be found that the so called lone electron pairs are gradually formed and become more dispersive with the weakened bonding nature between A–P interaction. In zintl phase, these lone electron pairs are located energetically close to the Fermi level, and there exists repulsion between them which is associated with increased band dispersion or destabilization of the corresponding bands. In addition, lattice vibrations, to some degree, will change the magnitude of repulsive lone pair interactions due to electron–phonon interaction, which can lead to a band shift of narrow bands (localized electrons) above the Fermi level resulting in an electron transfer from localized to delocalized bands. As the atomic mass of A site increases, the mass difference between A site and P atom enlarges resulting in stronger optical branch lattice vibration and repulsive interaction. The electron pairs between IA/IIA and P are often defined as nearly free electrons (NFE) while that between IIIA/IVA‐P are tight‐binding electrons (TBE).^[^ ^19^ ^]^ As the size and mass of A site cation improve, the NFE and repulsive effect enlarge, may be responsible for the degradation of band gap. However, it is still confusing that the introducing strongly electropositive alkali and alkaline earth metal element (Ca, Sr, Ba, Na, K, Rb, Cs) can promotes the band gap of chalcogenides but cannot work on pnictides.^[^ ^20^ ^]^

**Table 1:** Key information of the title compounds

Compound	Cal. *E*_g_ [eV]	Exp. *E*_g_ [eV]	Electronegativity of A site (Pauling scale)	Atomic radius of A site	*R*_0_ of A‐P bond	Maximum value of EDD field (*10‐)
MgSiP_2_	2.41	2.33	1.31	1.73	2.29	2.373
Li2SiP_2_	2.32	N/A	0.98	1.82	N/A	2.115
CdSiP_2_	2.13	2.2	1.69	2.18	2.34	2.092
CaSiP_2_	2.04	N/A	1.0	2.31	2.55	2.006
Na2SiP_2_	1.98	N/A	0.93	2.27	2.36	1.937
K2SiP_2_	1.86	N/A	0.82	2.75	2.64	1.768
Cs2SiP_2_	1.83	N/A	0.79	3.43	2.93	1.744
MgSiAs_2_	1.94	1.83	1.31	1.73	2.38	1.908
Rb2SiAs_2_	1.25	N/A	0.82	3.03	2.87	1.480
SrSiAs_2_	0.79	N/A	0.95	2.49	2.76	1.176

Herein, the idea of Zintl chemistry^[^ ^11^, ^21^ ^]^ was used to interpret this confusing band gap issue, which has been proved applicable and rational in plenty of Zintl compounds although it is a very simple chemistry concept. The physical behavior of an element is related to the valence electrons and orbitals used for chemical bonding. There is one less valence electron in P element than S element, which means the achievement of octet closed shell of P anions requires larger charge transfer. However, P anions which require larger charge transfer cannot stabilize that large charge without suitable cation environment, and on loosing close cation contacts they become highly reductive, i.e. “free some electrons” entering into structural space and causing the charge delocalization.^[^ ^22^ ^]^ Due to the size effect and electrostatic force interaction of IA/IIA cations, the P and S anions near them commonly only have one or two coordination numbers (CN). For IA/IIA metal sulfides, the octet electrons can be highly localized around S atoms due to its large electronegativity (*χ* = 2.58) and high electron affinity (200.41 kJ mol^−1^), so the band gap is enhanced. However, due to contractive electronegativity (*χ* = 2.2) and much lower electron affinity (72.04 kJ mol^−1^) of P atom, the nonbonding electron pairs of P atoms become delocalized. To form stable octet electronic configuration, P atoms need to form three TNE bonds (3s^2^ are generally inert) at least. It is commonly assumed that that chemical bonding in Zintl phase has a mixed ionic–covalent–metallic nature.^[^ ^23^ ^]^ Bonding within the polyanionic framework [SiP_2_]^2−^ using the Zintl formalism is considered localized, two‐center‐two‐electron bonding, whereas in the cases where the A‐P bond is not pure ionic interaction anymore, and it is better described as “polar intermetallics”^[^ ^19^ ^]^ when emphasizing that the extent of charge transfer from the cation to the polyanionic unit is incomplete, include delocalized multicenter bonding. In other words, the improvement of ionicity degrades the covalent degree of P atom resulting in “leakage electron” and the increase of metallicity. Therefore, the band gap cannot be effectively widened by the introducing of heavier IA/IIA metals. The reason why MgSiP_2_ and Li_2_SiP_2_ possess wider band gap among them is that both Mg and Li have somewhat covalent ability to P atoms and the electron pairs between Mg/Li‐P bonds tend to TBE. Structurally speaking, Mg and Li are electropositive element limits for diamond‐like compounds not breaking tetrahedral network due to their small size and certain covalent capability, e.g. diamond‐like compounds, MgSiP_2_, Mg_2_In_3_Si_2_P_7_, LiGaS_2_, and Li_4_Mg_2_GeS_7_.^[^ ^24^ ^]^ The Mg‐Pn and Li‐Pn bonds are dominated by polar covalent nature. Accordingly, MgSiP_2_ and Li_2_SiP_2_ exhibit wider band gap than CaSiP_2_, Na_2_SiP_2_, K_2_SiP_2_, and Cs_2_SiP_2_. In addition, under the formation of 4CN for P atom, MgSiP_2_ exhibits wider band gap than ZnSiP_2_ and CdSiP_2_ because the larger electropositivity of Mg (*χ*_Mg_ = 1.31, *χ*_Zn_ = 1.65, *χ*_Cd_ = 1.69) leads to a shift of the covalent bridge of the bond to the electronegative P atom. Our further investigation over other pnictides also showed that pnictides with P atoms of 2CN and 1CN generally have smaller band gap than those with P atoms of higher CN (Table S2, Supporting Information).

**Figure 5:**
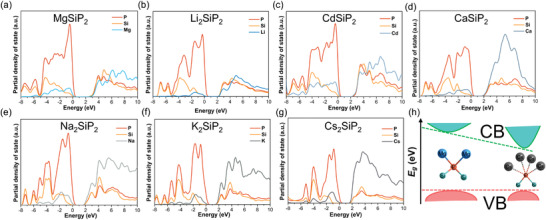
Partial density of states of MgSiP_2_ (a), Li_2_SiP_2_ (b), CdSiP_2_ (c), CaSiP_2_ (d), Na_2_SiP_2_ (e), K_2_SiP_2_ (f), and Cs_2_SiP_2_ (g). Schematic diagram of band gap enlargement (h).

To get insight into the atomistic origins of band gap, the partial density of states was systematically analyzed. The top of valence band for all title phosphides is mainly composed of P orbitals but the constitution of bottom of conduction band is distinguishing with the variety of A site atoms. In MgSiP_2_, Li_2_SiP_2_, and CdSiP_2_ (**Figure** **5**a‐c), the bottom of conduction band consists of Si, P, and Mg orbitals together. The orbitals of P and Si play a key role on the formation of conduction band edge (Figure S7b,f, Supporting Information) while those of Mg, Li, and Cd dominate on higher‐energy level conduction band attributed to the polar covalency of Mg/Li/Cd‐P bonds. Whereas, in Ca_2_SiP_2_, Na_2_SiP_2_, K_2_SiP_2_, and Cs_2_SiP_2_ (Figure 5d‐g), the A site orbitals make a leading contribution to the bottom of valence band, indicating the band edge is determined by the A–P interaction. This suggested that there is strong orbital coupling between A and P atoms, leading to a delocalized electron distribution across a long distance. Intriguingly, this is contrasting with alkali/alkaline earth metal NLO chalcogenides and oxides, where the orbitals of strongly ionic A site atom generally occupy higher energy level of conduction band, and the band gap mainly depend on anionic framework, and the wavefunction overlap of A cation and neighboring anion is negligible. Introducing alkali/alkaline earth metals to chalcogenides and oxides can effectively reduce the extent of orbital overlap in various directions in the lattice of the anionic framework, which leads to narrowing of the electronic bandwidths and widening of the band gaps.^[^ ^14^ ^]^ However, this “dimensional reduction’’ via introducing alkali/alkaline earth metals into pnictides does not widen band gap like chalcogenides and oxides. Actually, the A–P interaction in title phosphides is not ionicity anymore but “polar intermetallics” with more delocalized electron distribution, pushing the conduction band edge to a lower energy level. Figure S7 (Supporting Information) exhibits a detailed comparison of conduction band minimum (CBM) structure of MgSiP_2_ and CaSiP_2_, Li_2_SiP_2_ and K_2_SiP_2_, respectively. Compared with CaSiP_2_ and K_2_SiP_2_, MgSiP_2_ and Li_2_SiP_2_ have flatter energy band and higher energy level orbital contribution of A site atoms to CBM, which indicates more localized charge distribution in MgSiP_2_ and Li_2_SiP_2_ leading to their wide band gap (Figure 5h)

To further confirm our results, the electron density difference (EDD) analyses were performed, which can visualize the charge transfer and redistribution after the formation of all chemical bonds in the system. As is shown in (Figure 3e,f), besides Si‐P bonds, there is still strong covalent interaction formed between Mg/Cd/ and P atoms featuring high localized electrons, and In Li_2_SiP_2_ (Figure S5b, Supporting Information), two shorter Li–P bonds have relative covalent nature. However, as the atomic radius and mass of A site element increases, the electronic clouds of P atoms toward A site atoms gradually vanish (Figure 3g,h), meaning the electrons are more dispersive over the space between A and P atoms. In other words, the octet shell of P anion is no longer compact due to the disappearance of covalent interaction between A and P atoms with increasing polar intermetallics. As shown in Figure 3i,j, the electron distributions of MgSiP_2_ and CdSiP_2_ are remarkably more localized than those of Na_2_SiP_2_, K_2_SiP_2_, and Cs_2_SiP_2_ (Figure 3k,l, Figure S5c, Supporting Information). The maximum value of EDD fields gradually decreases from 0.2373 of MgSiP_2_ to 0.1744 of Cs_2_SiP_2_, also indicating the increase of electron delocalization (Table 1) and similar cases occur in the title arsenides (Figure S6, Supporting Information).

Consequently, to design band gap‐wide NLO pnictides, the ionic–covalent–metallic nature in system should be comprehensively tailored by suitable composition‐structure design. More importantly, this principle uncovered that the difficult balance between band gap and second‐order NLO performance may be better achieved in NLO pnictides because the enhanced covalent nature can concurrently elevate these two competing NLO criteria. For instance, the increasing coordination numbers of P atom lead to increasing density of [MP4] tetrahedral group, which might contribute to NLO coefficient when these tetrahedra are optimally arranged. MgSiP_2_ is a successful example which well balances the ionicity–covalency and tremendously decrease the metallicity by Mg‐P polar covalent bond. Moreover, its chalcopyrite structure with aligned [MgP_4_] and [SiP_4_] tetrahedron units are favorable to positive geometric superposition of microscopic second‐order NLO susceptibility leading to strong macro‐SHG effect. It was first reported in 1969 and published in *Nature* as an important inorganic chemistry discovery.^[^ ^10d^ ^]^ Over the past decades, its optical and other physical properties based on experiment were rarely investigated due to its high melting point and difficult crystal growth.^[^ ^25^ ^]^ In this work, we successfully synthesized millimeter‐level crystals of MgSiP_2_ using BaCl_2_ salt flux and measured its IR transparency and nonlinear optical properties for the first time.

**Figure 6:**
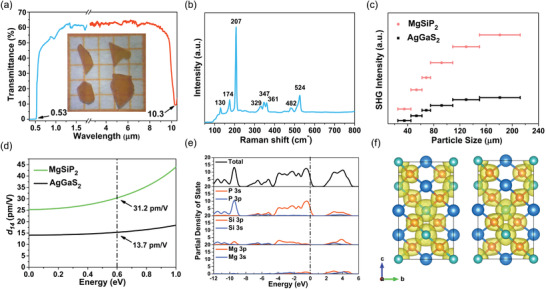
UV‐vis‐IR transmittance spectrum (a) with crystal wafer images of MISP (inset in a), one‐phonon Raman scattering spectrum (b), particle size‐dependent SHG intensity curves with references of AgGaS_2_ (c), Calculated frequency‐dependent SHG coefficients (d), calculated refractive index dispersion curves (e), and SHG density map of occupied state and unoccupied state (f).

Its purity were confirmed through powder XRD diffraction (Figure S9, Supporting Information). The elemental stoichiometry of Mg:Si:P was roughly 1:1:2 according to energy‐dispersive spectrometry analysis (Figure S10, Supporting Information). The vis−IR transmittance spectrum (**Figure** **6**a) was measured based on 0.5 mm‐thick crystal wafers. The short wave transparency cut‐off located on 0.53 µm corrensponding to the band gap of 2.34 eV. Notably, attributed to the optimal ionic–covalent‐metallic nature of MgSiP_2_, it successfully broke through the “2.33 eV wall” avoiding two‐photon absorption for 1 µm laser pump. The IR transparency cut‐off is at 10.3 µm, which is comparable to that of CdSiP_2_ and ZnSiP_2_ (≈10 µm).^[^ ^26^ ^]^ The long‐wave IR edge of MgSiP_2_ is determined by two‐phonon absorption resulting from high frequency vibration of Si−P bonds. The one‐phonon Raman scattering spectrum (Figure 6b) was measured to analyze the vibration distribition of atoms and chemical bonds. The high frequency peaks were mainly ascribed to the vibrations of stiff Si−P bonds, while the low frequency peaks mainly arose from the vibrations of soft Mg−P bonds. The highest frequency vibration of Si−P bonds at 524 cm^−1^ corresponds to the IR absorption edge of 10.3 µm (971 cm^−1^) under two‐phonon absorption approximation of diamond‐like compounds. The TG/DTA curves (Figure S11, Supporting Information) showed MgSiP_2_ were stable up to 870 °C without decomposition, indicating its excellent thermostability.

**Figure 7:**
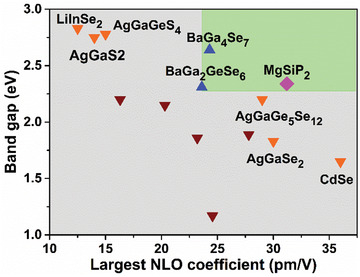
Two‐dimensional diagram presenting the balanced performance of the SHG response and band gap. The benchmark is BaGa_2_GeSe_6_ with band gap of 2.31 eV and *d*_11_ of 23.6 pm V^−1^.^[^ ^29^ ^]^ The green area represents a high balance of SHG response and band gap and the gray area is opposite. The red and orange triangle symbols represent some recently reported and the state‐of‐the‐art NLO chalcogenides, respectively.

The NLO performance was characterized by Kurtz and Perry method with AgGaS_2_ as the references.^[^ ^27^ ^]^ The particle size‐dependent SHG intensity curve of MgSiP_2_ (Figure 6c) revealed that it is phase‐matchable under 2050 nm laser irradiation, and exhibited strong SHG effects which were 3.5 times that of AgGaS_2_. The excellent second‐order NLO performance of MgSiP_2_ orginates from the arrangement‐aligned [SiP_4_] and [MgP_4_] tetrahedra with high structure criterion *C* value of 0.985 (Table S3, Supporting Information). Under the restriction of Kleinman's symmetry, MgSiP_2_ only has one nonzero independent NLO coefficient (Figure 3a,b), namely, *d*_14_, which was 31.2 pm V^−1^ at 2050 nm (0.60 eV). The *d*_14_ of MgSiP_2_ was ≈2 times that of AgGaS_2_ (*d*_14_ = 13.7 pm V^−1^) and comparable to that of AgGaSe_2_ (*d*_14_ = ≈30 pm V^−1^).^[^ ^28^ ^]^ As shown in **Figure** **7**, MgSiP_2_ presents superior balanced performance of optical nonlinearity and band gap in comparison with the state‐of‐the‐art and recently reported NLO chalcogenides, comparable to BaGa_4_Se_7_ and BaGa_2_GeSe_6_ (see details in Table S4, Supporting Information). The PDOS plots (Figure 6e) showed that the top of valence band consisted of P 3p orbitals while the bottom of conduction band was mainly dominated by 3p orbitals of P, Si, and Mg. The SHG density patterns (Figure 6f) showed that the SHG effect mainly originated from [SiP_4_] tetrahedron units. The atomic contribution to the SHG coefficient based on Bader charge analysis showed that Mg, Si, and P have contributions of 5.7%, 30.5%, and 63.8% to *d*_14_, respectively, which indicates nonignorable effect of [MgP_4_] tetrahedra.

### 2.1 Conclusion and Outlook

In summary, we unveiled an important and general band gap mechanism of pnictides, i.e. P atom with low coordination numbers (2 CN) will decrease band gap due to the delocalization of nonbonding electron pairs. A tactic for the rational design of wide‐band gap NLO pnictides, ionicity–covalency–metallicity regulation, was proposed for achieving the desired balance between the band gap and SHG effect. Guided by this, millimeter‐level MgSiP_2_ single crystal was grown via salt‐flux BaCl_2_. MgSiP_2_ exhibited a largest band gap of 2.34 eV among chalcopyrite pnictides overcoming the two‐photon absorption under 1 µm laser irradiation, a strong NLO performance (3.5 x AgGaS_2_) and a wide IR transparency range (0.53–10.3 µm). Furthermore, the ionic–covalent–metallic nature control strategy opens a new avenue for obtaining NLO pnictides with balanced band gap and NLO performance. This provides a future exploration direction i.e. designing the structure with P atom of high coordination number (>2CN), or introduce more electronegative chalcogens and halogens to pnictides for undertaking part transferring charge and promoting the octet shell stabilization. This work may also gives some inspiration on other Zintl phase for thermoelectric, superconductive, and transport materials.

## 3 Experimental Section

### Reagent

Mg (Adamas, 4N), Si (Aladdin, 4N), P (Taitan, 5N), and BaCl_2_ (Adamas, 3N)

### Synthesis

Reactants of Mg, Si, P, and flux BaCl_2_ with a molar ratio of 1.33/1/2/2.67 were thoroughly grinded and mixed in a glove box under an Ar gas atmosphere. The mixture was loaded into a graphite crucible, then flame‐sealed into a quartz tube. Subsequently, the tube was placed in a muffle furnace, and heated to 1100 °C, holding for 3 days, and slowly cooled to 800 °C at a rate of 3 °C h^−1^, finally cooled to 300 °C at a rate of 10 °C h^−1^ before naturally cooling. The air‐ and moisture‐stable yellow crystal grains (90% yield) were obtained and then washed using deionized water to remove BaCl_2_. Some millimeter single crystals were picked out and processed into crystal wafer for further measurements.

### Powder X‐ray Diffraction and Microprobe Elemental Analysis

Powder XRD pattern of polycrystalline materials were obtained on a Miniflex‐600 powder X‐ray diffractometer with Cu K*α* radiation (*λ* = 1.540 59 Å) at room temperature within an angular range of 2*θ* = 10−80° with a scan step width of 0.02° and a fixed time of 0.2 s. Microprobe elemental analysis were performed on a field emission scanning electron microscope (SU‐8010) equipped with an energy‐dispersive X‐ray spectrometer.

### Single Crystal X‐Ray Diffraction

The diffraction data were collected at room temperature on a Rigaku Oxford Hybrid Pixel Array diffractometer (Liquid MetalJet D2+) with mirror‐monochromatic Ga K_*α*_ radiation (*λ* = 1.34050 Å) and *ω*‐scan technique was used to correct Lorentz and polarization factors. The data was integrated with CrysAlisPro program and the multiscan method was used to correct the absorption.

### Thermal Analysis

Thermal analysis (DTA) were performed on a NETZSCH STA 449F3 unit in N2 atmosphere at 10 °C min^−1^ heating rate. An amount of 20 mg of title compounds were ground into fine powder and enclosed in Al_2_O_3_ crucible. The well‐prepared samples were heated from room temperature to 1100 °C at a rate of 10 °C min^−1^.

### Optical Properties

The UV−vis−NIR transmittance spectrum from 250 to 2500 nm and the IR transmittance spectra from 4000 to 400 cm^−^ based on polished crystal wafers were recorded using Lamda‐950 UV/Vis/NIR spectrophotometer Lamda‐950 UV/Vis/NIR spectrophotometer and Bruker VERTEX 70 FTIR spectrophotometer, respectively. Polycrystalline second harmonic generation (SHG) measurements were measured through the Kurtz−Perry method^[^ ^27^ ^]^ with Q‐switched Nd: YAG solid‐state laser having a wavelength of 2050 nm. The samples were ground thoroughly and then sieved into six distinct particle size ranges of 25−45, 45−62, 62−75, 75−109, 109−150, and 150−212 µm. Crystalline AGS with the same particle size ranges were prepared as the references.

### Computational Methods

The first‐principles calculation was conducted using a plane‐wave pseudopotential package CASTEP based on density functional theory (DFT).^[^ ^30^ ^]^ The exchange‐correlation energy was described by the generalized gradient approximation (GGA) scheme of Perdew−Burke−Ernzerhof (PBE) functional.^[^ ^31^ ^]^ Norm‐conserving pseudopotentials^[^ ^32^ ^]^ (Mg 2s^2^sp^6^3s^2^, Si 3s^2^3p^2^, P 3s^2^3p^3^) were employed to simulate the ion‐electron interactions. The kinetic energy cutoff is set to be 990 eV for MgSiP_2_. Monkhorst–Pack^[^ ^33^ ^]^ k‐point meshes of 4 × 4 × 2 spanning less than 0.04 Å^−3^ in the Brillouin zone were chosen. The unit cells were fully optimized using BFGS^[^ ^34^ ^]^ method before electronic structure calculations. Due to the discontinuity of exchange‐correlation, band gaps calculated by the GGA method are usually smaller than experimental values. Therefore, a scissors operator^[^ ^35^ ^]^ was adopted to raise the conduction bands to match the experimental value. Using the scissors‐corrected electronic structure, the second‐order nonlinear susceptibility was calculated through the “velocity‐gauge” formula, and the SHG density was calculated by a band‐resolved method.^[^ ^36^ ^]^ The HOMO‐LUMO gap, polarizability anisotropy, and hyperpolarizability of anionic groups (CdP_4_)^10−^, (GaP_4_)^9−^, (GeP_4_)^8−^, (SiP_4_)^8−^, (ZnS_4_)^6−^, and (GaS_4_)^5−^ were calculated using DFT implemented by the Gaussian 09 package. The basis set LanL2DZ with B3LYP (Becke, three‐parameter, Lee–Yang–Parr) exchange‐correlation functional was employed. The HSE06 exchange‐correlation functional^[^ ^16^ ^]^ was applied to calculate the precise band gap of A^II^SiPn_2_ and A^I^_2_SiPn_2_ (A^II^ = Cd, Mg, Ca, and Sr; A^I^ = Li, Na, K, Rb, and Cs; Pn = P and As). The k‐points of Monkhorst–Pack grid used in the calculation of MgSiP_2_, MgSiAs_2_, CdSiP_2_, CaSiP_2_, SrSiAs_2_, Li_2_SiP_2_, Na_2_SiP_2_, K_2_SiP_2_, Rb_2_SiAs_2_, and Cs_2_SiP_2_ were 4 × 4 × 2, 4 × 4× 2, 4 × 4× 2, 3 × 3 × 1, 2 × 2 × 2, 2 × 2 × 3, 2 × 1 × 4, 2 × 4 × 4, 3 × 2 × 4, and 2 × 3 × 4, respectively. The ELF (*η*)^[^ ^18^ ^]^ and charge density differece was evaluated with modules implemented within the Materials Studio software. The EDD reflect the difference between the bonded charge density and the atomic charge density at the corresponding point, which is expressed as Δ*ρABC* = *ρABC* − *ρA* − *ρB* − *ρC*, *ρABC* represents the charge density of ABC system after the formation of the chemical bonds, *ρ*_A_,*ρ*_B_,and *ρ*_C_ are the charge density of ideal A, B, and C atoms, respectively.^[^ ^37^ ^]^ Through the analysis of charge density difference, the charge movement and transfer in the process of bonding and bonding electron coupling can be clearly observed. This method was used to quantitatively analyze the real‐space charge distribution on the octet shell of P atom in system.

## Conflict of Interest

The authors declare no conflict of interest.

## Supporting information

Supporting Information

Supporting Information

## Data Availability

Research data are not shared.
