# Supplementary material for: Uncovering a Vital Band Gap Mechanism of Pnictides
Source: Adv Sci (Weinh). 2022 Mar 31;9(14):2105787. doi: 10.1002/advs.202105787 (PMC9109059; doi:10.1002/advs.202105787)
Supplement: Supplementary file 1 — Supporting Information [file ADVS-9-2105787-s001.pdf]

ADVANCED SCIENCE
Open Access

Supporting Information

for *Adv. Sci.*, DOI 10.1002/advs.202105787

Uncovering a Vital Band Gap Mechanism of Pnictides

Jindong Chen, Qingchen Wu, Haotian Tian, Xiaotian Jiang, Feng Xu, Xin Zhao, Zheshuai Lin, Min Luo and Ning Ye**

Supporting Information

© 2022 Wiley-VCH GmbH
69451 Weinheim, Germany

Uncovering a Vital Band Gap Mechanism of Pnictides

Jindong Chen,[a], [e] Qingchen Wu,[c] Haotian Tian,[a] Xiaotian Jiang,[d] Feng Xu,[a] Xin Zhao,[a] Zheshuai Lin,[c] Min Luo[a]* and Ning Ye[a], [b]*

Abstract: Pnictides are superior IR NLO material candidates, but the exploration of NLO pnictides is still tardy due to lack of rational material design strategies. An in-depth understanding structure-performance relationship is urgent for designing novel and eminent pnictide NLO materials. Herein, we unraveled a vital band gap mechanism of pnictides, namely P atom with low coordination numbers (2 CN) will cause the decrease of band gap due to the delocalization of non-bonding electron pairs. Accordingly, a general design paradigm for NLO pnictides, ionicity–covalency–metallicity regulation was proposed for designing wide-band gap NLO pnictides with maintained SHG effect. Driven by this idea, millimeter-level crystals of MgSiP2 were synthesized with a wide band gap (2.34 eV), a strong NLO performance (3.5 x $AgGaS_2$) and a wide IR transparency range (0.53-10.3 μm). This work provides an essential guidance for the future design and synthesis of NLO pnictides, and also opens a new perspective at Zintl chemistry important for other material fields.

Table of Contents

Table S1. Crystallographic Data and Refinement Details for $MgSiP_2$.

Formula	$MgSiP_2$
Formula weight	114.34
Temperature/K	293
Radiation	Ga K_{α} (λ = 1.34139)
Crystal system	tetragonal
Space group	I-42d
a/Å	5.7156(5)
c/Å	10.074(2)
α/°	90
β/°	90
γ/°	90
Volume/Å^3	329.11(10)
Z	4
ρcalc (g/cm^3)	2.308
μ (mm-1)	9.737
F (000)	224
Index ranges	$-5 \le h \le 3$, $-7 \le k \le 6$, $-11 \le l \le 12$
Reflections collected	395
Independent reflections	175 [R_{int} = 0.0141
Data/restraints/parameters	175/0/11
Goodness-of-fit on F^2	1.212
Final R indexes [I>=2σ (I)]	R_1 = 0.0328, wR_2 = 0.0862
Final R indexes [all data]	R_1 = 0.0330, wR_2 = 0.0866
Largest diff. peak/hole / e Å^{-3}	0.596/-0.659
Flack parameter	0.1(5)

Table S2. Th relationship between the coordination number of P atom and the band gap of phosphides.

Compounds	Coordination number of P atom (CN)	Band gap (eV)	Reference
Zn_3PI_3	4	2.85	1
Cd_2PCl_2	4	>2.5	2
Cd_3PI_3	4	2.44	1
GaP	4	2.4	3
$MgSiP_2$	4	2.33	4
$Cd_4P_2Cl_3$	4	2.36	5
$CdSiP_2$	4	2.2	6
α-ZnP_2	4	2.25	7
α-CdP_2	4	2.0	8
$IrSi_3P_3$	4	1.93	9
$RuSi_4P_4$	4	1.83	9
$NaGe_3P_3$	3	2.06	10
SiP (monolayer)	3	2.59	11
$Ba_2Si_3P_6$	2 and 3	1.88	12
KSi_2P_3	2 and 3	1.72	13
$BaSi_7P_{10}$	2 and 3	1.1	14
$SrSi_7P_{10}$	2 and 3	1.1	14
Ba_2SiP_4	2	1.45	15

Sr_2SiP_4	2	1.41	15
$BaGe_2P_2$	2	1.36	16
$LaSiP_3$	1, 2 and 3	1.3	17
β-Ca_2CdP_2	1, 2 and 3	1.55	18

Table S3. Calculated values of g_{ijk} and *C*.

Compouds	Groups	g_{ijk}	*C*
	$[ZnP_4]$	g_{123} = 0.99950	0.99949
$ZnGeP_2$	$[GeP_4]$	g_{123} = 0.99947	
	$[AgS_4]$	g_{123} = 0.98764	0.98513
$AgGaS_2$	$[GaS_4]$	g_{123} = 0.98263	
	$[MgP_4]$	g_{123} = 0.98653	0.98521
$MgSiP_2$	$[SiP_4]$	g_{123} = 0.98389	

Table S4. Comparison of $MgSiP_2$ with famous and recently reported NLO chalcogenides.

Compounds	E_g (eV)	d_{ij} (pm/V)	Reference
$MgSiP_2$	2.34	31.2	this work
$AgGaS_2$	2.75	14	19
$AgGaSe_2$	1.83	30	19
$BaGa_4Se_7$	2.64	20.6	20
$LiInSe_2$	2.83	12.5	21
$BaGa_2GeSe_6$	2.31	23.6	19
$AgGaGeS_4$	2.78	15	22
$AgGaGe_5Se_{12}$	2.2	29	23
CdSe	1.65	36	24
m-Ga_2Se_3	1.86	23.2	25
$Sr_6Cd_2Sb_6O_7S_{10}$	1.89	27.8	26
$SnI_4\cdot(S_8)_2$	2.17	6.3	27
$Na_2CdSn_2Se_6$	2.15	20.3	28
$Ba_6In_6Zn_4Se_{19}$	2.2	16.3	29
Hg_2GeSe_4	1.17	24.56	30

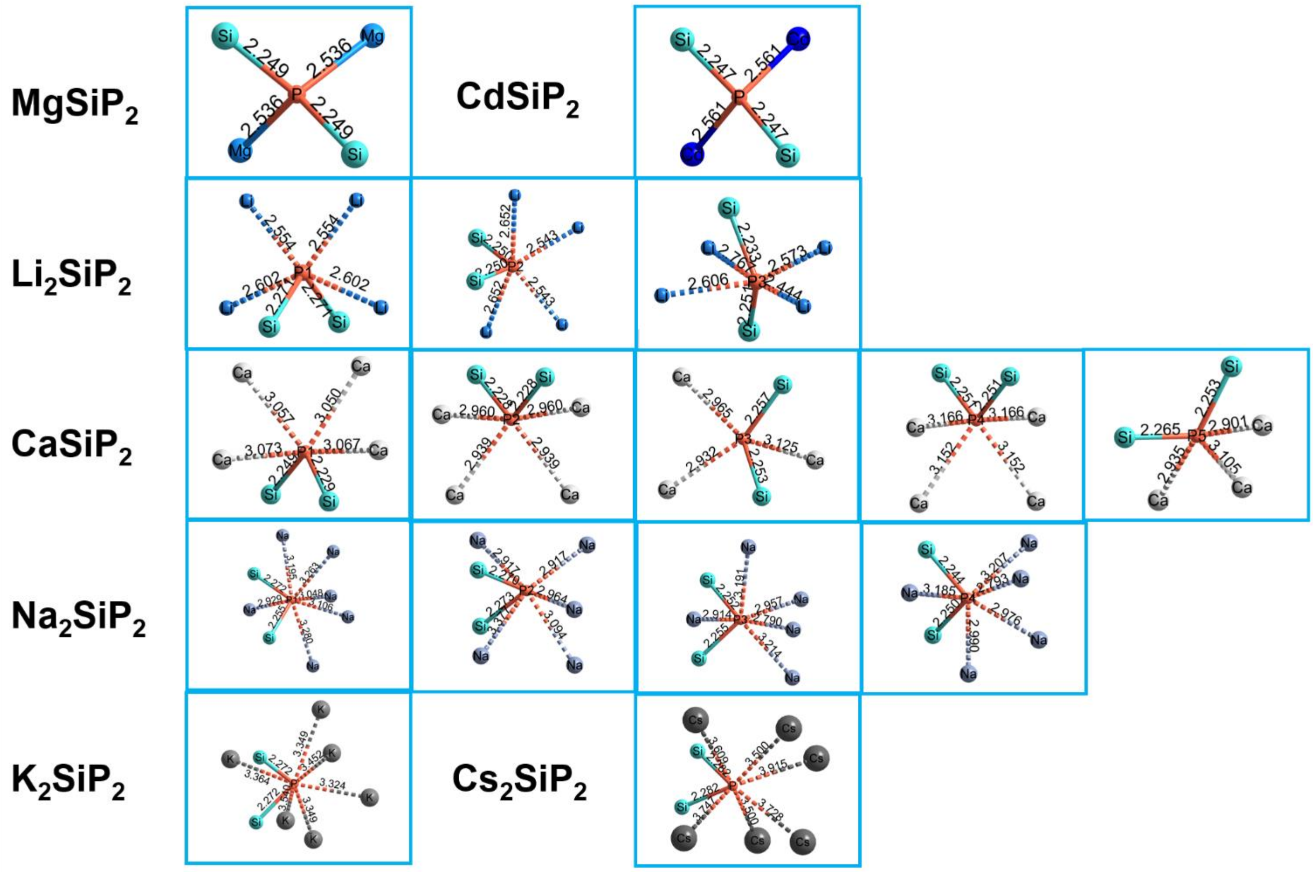

Figure S1. Coordination environment of P atoms.

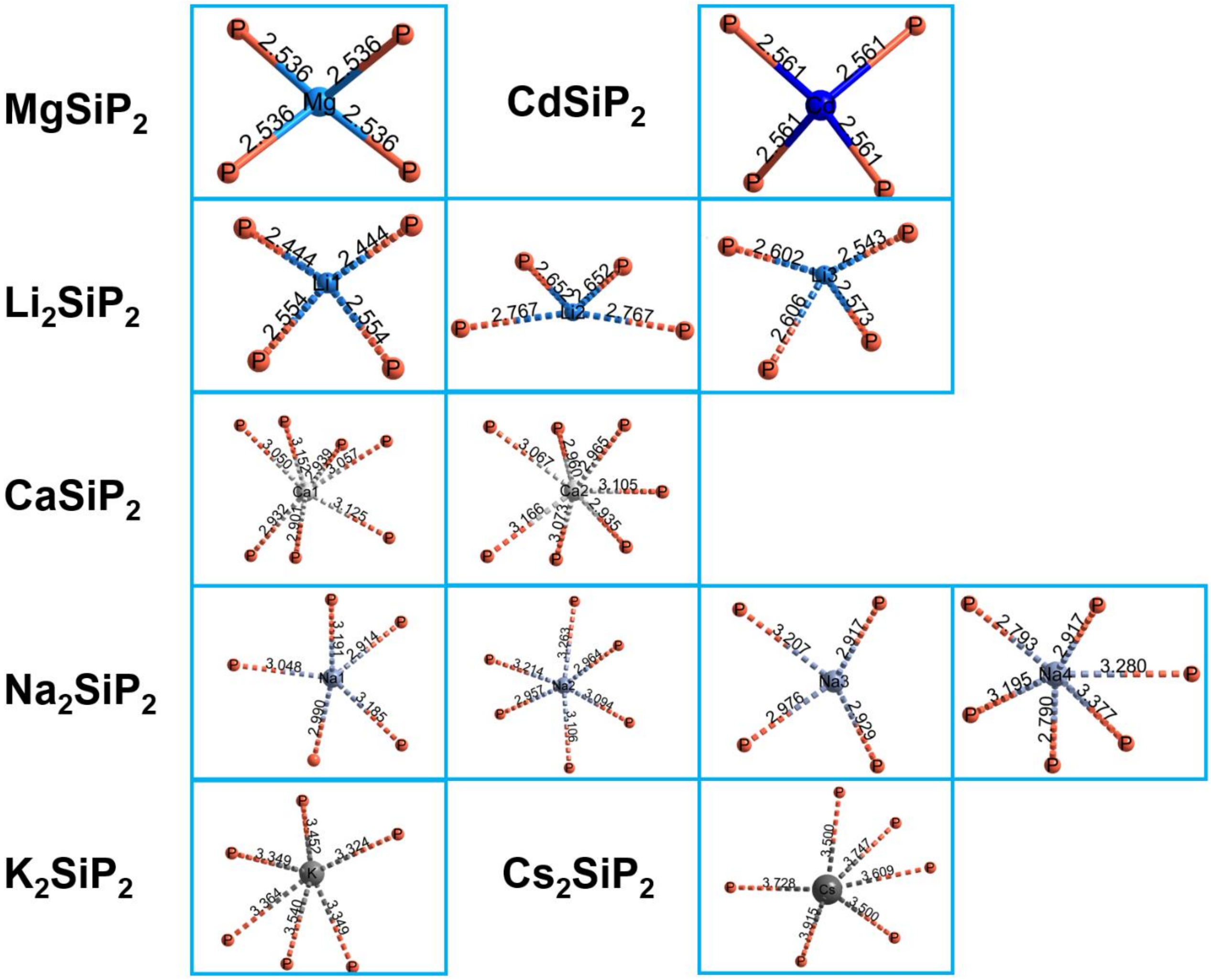

Figure S2. Coordination environment of A site atoms.

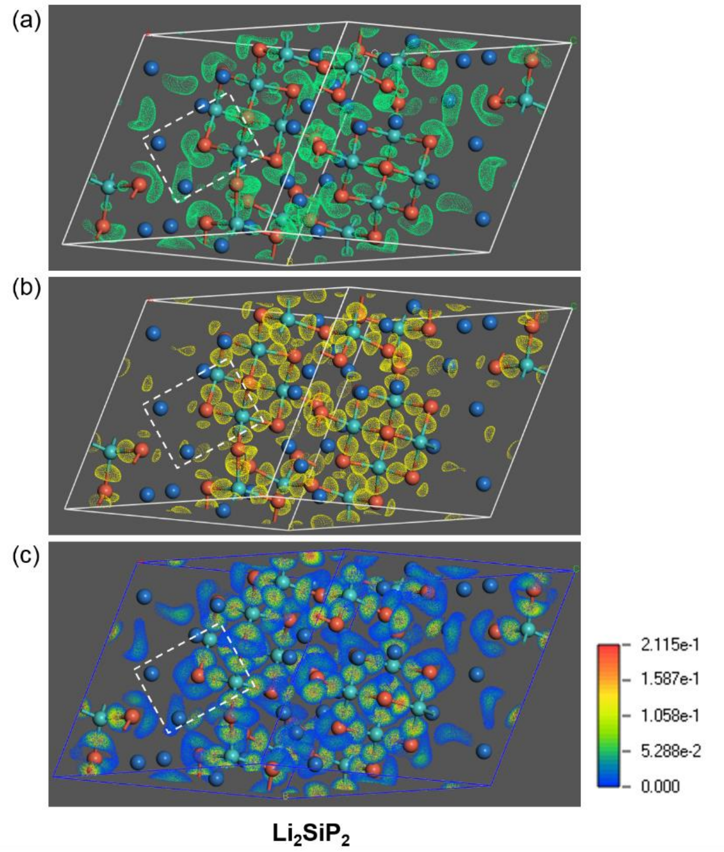

Figure S3. 3D ELF isosurfaces at η = 0.5 (a), EDD isosurfaces at η = 1/2 × maximum (b) and EDD distributions (c) of Li_2SiP_2. White dashed boxes represent two nearest Si and Li atoms of P atom.

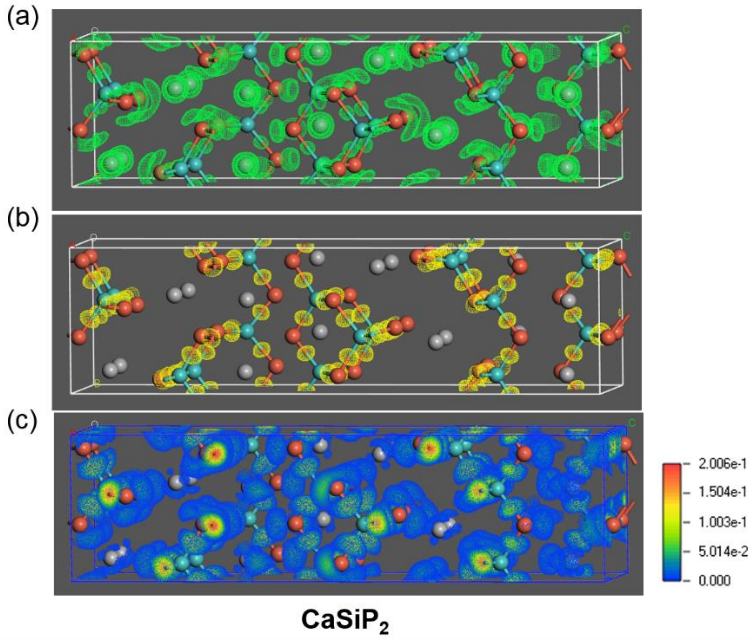

Figure S4. 3D ELF isosurfaces at η = 0.5 (a), EDD isosurfaces at η = 1/2 × maximum (b) and EDD distributions (c) of $CaSiP_2$.

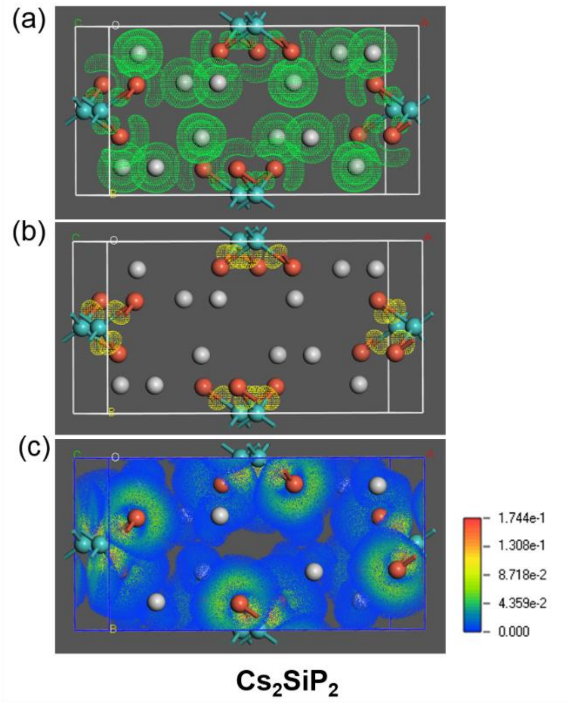

Figure S5. 3D ELF isosurfaces at η = 0.5 (a), EDD isosurfaces at η = 1/2 × maximum (b) and EDD distributions (c) of Cs_2SiP_2.

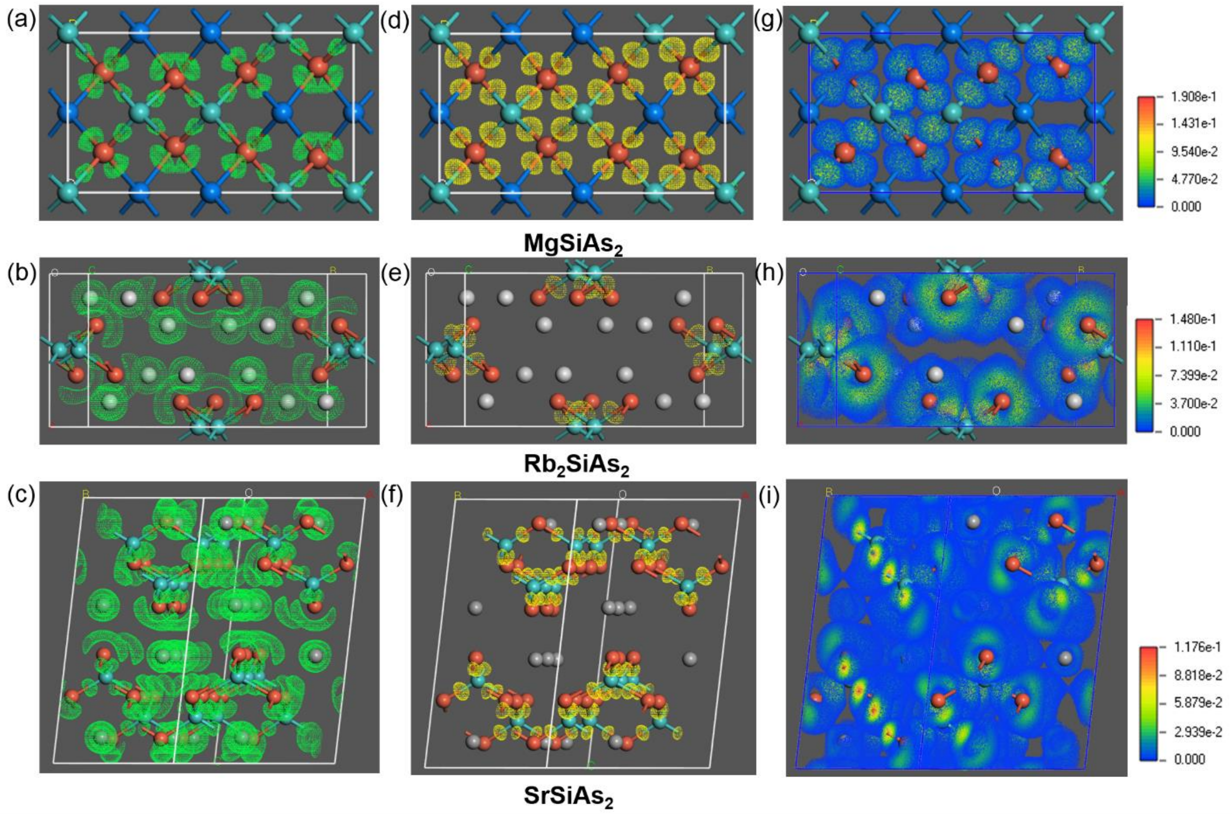

Figure S6. 3D ELF isosurfaces at η = 0.5 (left column), EDD isosurfaces at η = 1/2 × maximum (middle column) and EDD distributions (right column) of $MgSiAs_2$ (a, d and g), $RbSiAs_2$ (b, e and h), $SrSiAs_2$ (c, f and i)

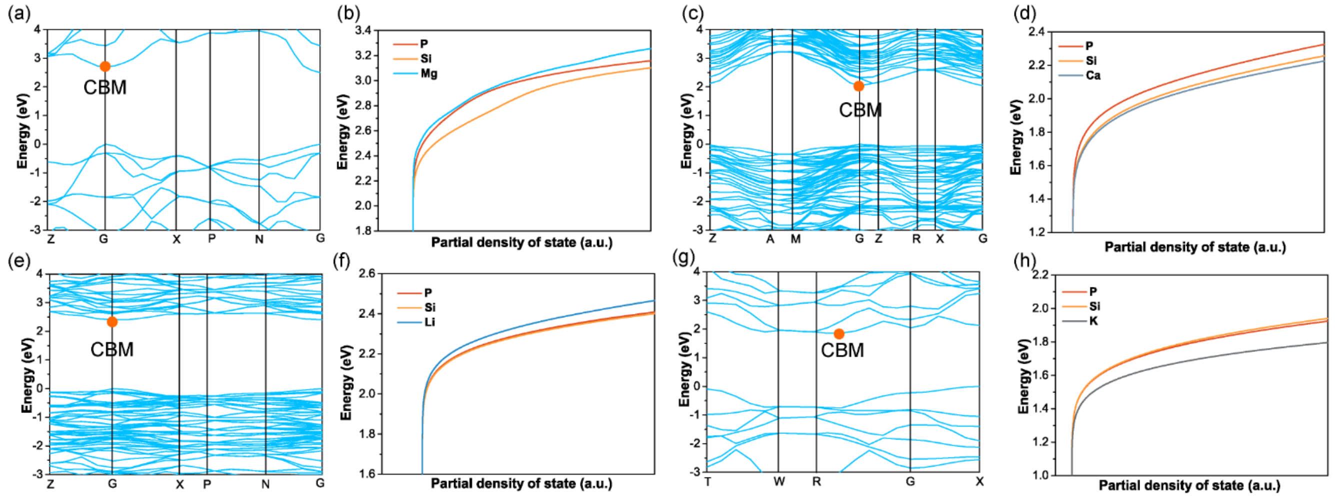

Figure S7. CBM structure comparison of $MgSiP_2$ (a, b) and $CaSiP_2$ (c, d), Li_2SiP_2 (e, f) and K_2SiP_2 (g, h).

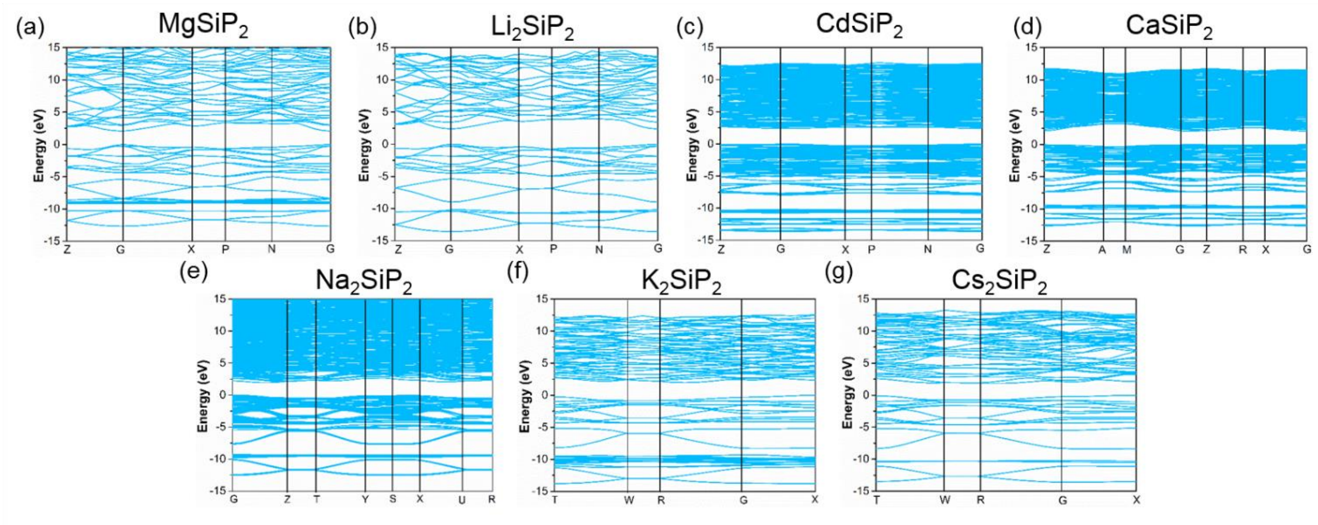

Figure S8. Band structure of $MgSiP_2$ (a), Li_2SiP_2 (b), $CdSiP_2$ (c), $CaSiP_2$ (d), Na_2SiP_2 (e), K_2SiP_2 (f) and Cs_2SiP_2 (g).

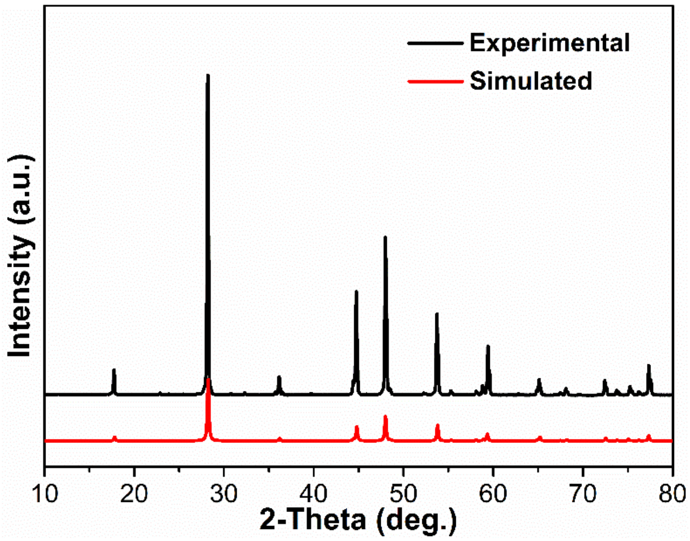

Figure S9. Powder XRD patterns of the experimental and simulated for $MgSiP_2$.

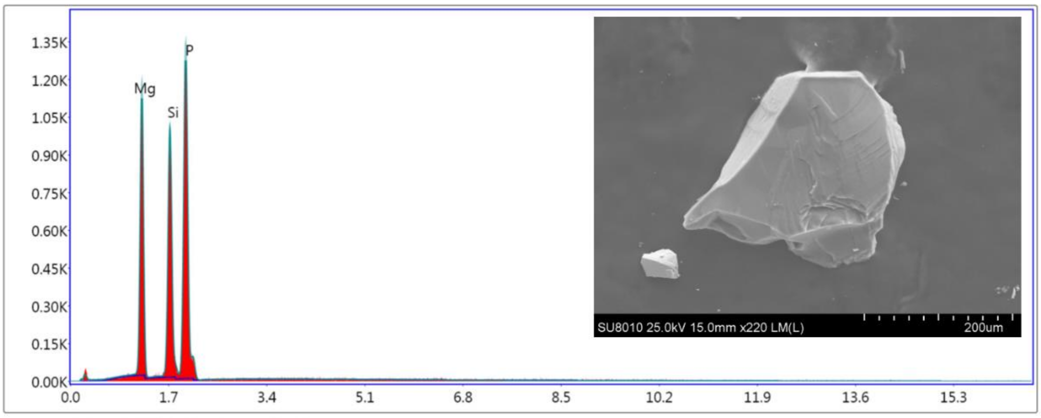

Lsec: 12.4 0 Cnts 0.000 keV Det: Octane Plus Det

Element	Weight %	Atomic %	Net Int.	Error %	Kratio	Z	R	A	F
MgK	21.64	25.44	1,472.40	4.19	0.17	1.03	0.98	0.75	1.02
SiK	23.50	23.92	1,447.24	5.32	0.16	1.02	1	0.65	1.03
P K	54.87	50.64	2,151.56	6.35	0.28	0.98	1.01	0.51	1

Figure S10. Energy-dispersive X-ray Spectroscopy analysis of $MgSiP_2$.

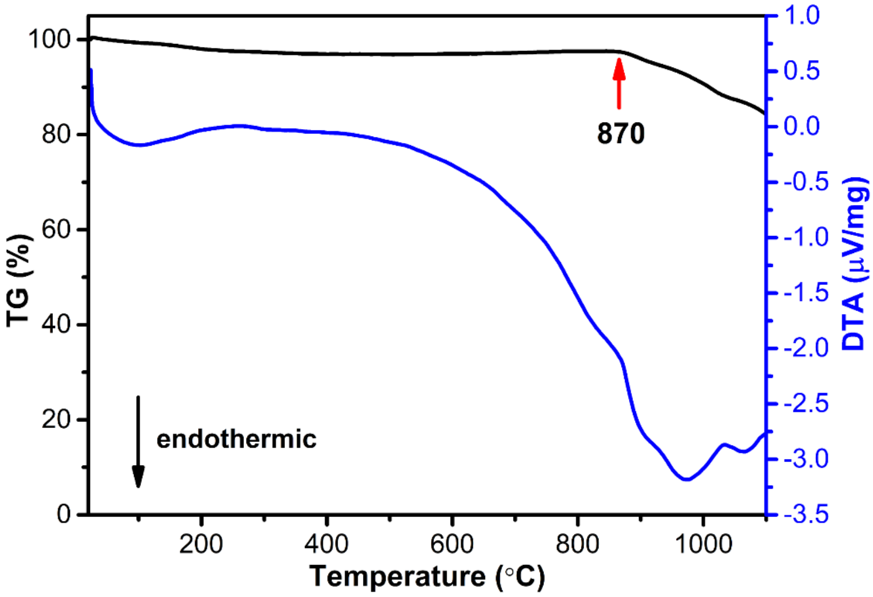

Figure S11. TG-DTA curves of $MgSiP_2$.

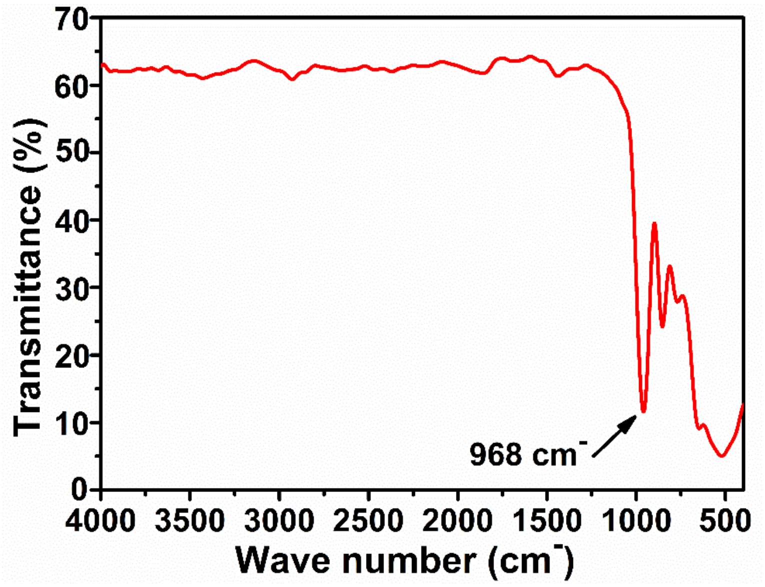

Figure S12. IR transmittance spectrum of $MgSiP_2$.

Reference

1. J. Chen, C. Lin, D. Zhao, M. Luo, G. Peng, B. Li, S. Yang, Y. Sun, N. Ye, *Angew. Chem., Int. Ed.* **2020**, *59*, 23549-23553.
2. A. V. Olenev, O. S. Oleneva, A. V. Shevelkov, B. A. Popovkin, *Russian Chemical Bulletin* **2003**, *52*, 570-575.
3. P. G. Schunemann, K. T. Zawilski, L. A. Pomeranz, D. J. Creeden, P. A. Budni, *J. Opt. Soc. Am. B* **2016**, *33*, D36-43
4. G. AmbrazeviD ius, G. Babonas, Y. V. Rud, A. E ileika, *Phys. Stat. Sol.* **1981**, *106*, 85-89
5. A. Roy, U. S. Shenoy, K. Manjunath, P. Vishnoi, U. V. Waghmare, C. N. R. Rao, *J. Phys. Chem. C* **2016**, *120*, 15063-15069.
6. G. Zhang, L. Wei, L. Zhang, X. Wang, B. Liu, X. Zhao, X. Tao, *J. Cryst. Growth* **2017**, *473*, 28-33.
7. I. E. Zanin, K. B. Aleinikova, M. Y. Antipin, *Crystallography Reports* **2003,** *48*, 199-204.
8. J. Chen, C. Lin, F. Xu, S. Yang, Y. Sun, X. Zhao, X. Jiang, B. Li, T. Yan, N. Ye, *Chem. Mater.* **2020,** *32*, 10246-10253.
9. S. Lee, S. L. Carnahan, G. Akopov, P. Yox, L. L. Wang, A. J. Rossini, K. Wu, K. Kovnir, *Adv. Funct. Mater.* **2021,** *31* (16), 2010293.
10. K. Feng, W. Yin, R. He, Z. Lin, S. Jin, J. Yao, P. Fu, Y. Wu, *Dalton Trans* **2012,** *41*, 484-489.
11. S. Zhao, P. Luo, S. Yang, X. Zhou, Z. Wang, C. Li, S. Wang, T. Zhai, X. Tao, *Adv. Opt. Mater.* **2021,** *9*, 2100198.
12. J. Mark, J. Wang, K. Wu, J. G. Lo, S. Lee, K. Kovnir, *J. Am. Chem. Soc.* **2019**, *141*, 11976-11983.
13. K. Feng, L. Kang, W. Yin, W. Hao, Z. Lin, J. Yao, Y. Wu, *J. Solid State Chem.* **2013**, *205*, 129-133.
14. A. Haffner, V. Weippert, D. Johrendt, *Z. Anorg. Allg. Chem.* **2020**, *647*, 326-330.
15. J. Mark, J.-A. Dolyniuk, N. Tran, K. Kovnir, *Z. Anorg. Allg. Chem.* **2019**, *645*, 242-247.
16. J. Chen, C. Lin, G. Peng, F. Xu, M. Luo, S. Yang, S. Shi, Y. Sun, T. Yan, B. Li, N. Ye, **2019**, *31*, 10170-10177.
17. Y. Sun, J. Chen, S. Yang, B. Li, G. Chai, C. Lin, M. Luo, N. Ye, *Adv. Opt. Mater.* **2021,** *9*, 2002176.
18. Y. Sun, C. Lin, J. Chen, F. Xu, S. Yang, B. Li, G. Yang, M. Luo, N. Ye, *Inorg. Chem.* **2021,** *60*, 7553-7560.
19. G. C. Catella, D. Burlage, *MRS Bulletin.* **2013,** *23*, 28-36.
20. X. Zhang, J. Yao, W. Yin, Y. Zhu, Y. Wu, C. Chen, *Opt. Express* **2015,** *23*, 552-558.
21. L. I. Isaenko, I. G. Vasilyeva, *J. Cryst. Growth* **2008**, *310*, 1954-1960.
22. K. Kato, V. V. Badikov, L. Wang, V. L. Panyutin, K. V. Mitin, K. Miyata, V. Petrov, *Opt. Lett.* **2020,** *45*, 2136-2139.
23. W. Huang, Z. He, S. Zhu, B. Zhao, B. Chen, S. Zhu, *Inorg. Chem.* **2019,** *58*, 5865-5874.
24. G. D. Boyd, E. Buehler, F. G. Storz, *Appl. Phys. Lett.* **1971,** *18*, 301-304.

25. Q.-T. Xu, Z.-D. Sun, Y. Chi, H.-G. Xue, S.-P. Guo, *J. Mater. Chem. C* **2019,** *7*, 11752-11756.
26. R. Wang, F. Liang, F. Wang, Y. Guo, X. Zhang, Y. Xiao, K. Bu, Z. Lin, J. Yao, T. Zhai, F. Huang, *Angew. Chem., Int. Ed.* **2019,** *58*, 8078-8081.
27. S. P. Guo, Y. Chi, H. G. Xue, *Angew. Chem., Int. Ed.* **2018,** *57*, 11540-11543.
28. R. Ye, X. Cheng, B.-W. Liu, X.-M. Jiang, L.-Q. Yang, S. Deng, G.-C. Guo, *J. Mater. Chem. C* **2020,** *8*, 1244-1247.
29. A. Zhou, C. Lin, B. Li, W. Cheng, Z. Guo, Z. Hou, F. Yuan, G.-L. Chai, *J. Mater. Chem. C* **2020,** *8*, 7947-7955.
30. Y. Guo, F. Liang, J. Yao, Z. Lin, W. Yin, Y. Wu, C. Chen, *Inorg. Chem.* **2018,** *57*, 6795-6798.
